# Zinc as a master regulator of intracellular organelle homeostasis

**DOI:** 10.1038/s12276-026-01706-2

**Published:** 2026-05-01

**Authors:** Sofia Brito, Jiyoon Kim, Bum-Ho Bin

**Affiliations:** 1 Department of Pharmacology, College of Medicine, The Catholic University of Korea, Seoul, Republic of Korea; 2 Department of Medical Sciences, Graduate School, The Catholic University of Korea, Seoul, Republic of Korea; 3 Institute for Aging and Metabolic Diseases, College of Medicine, The Catholic University of Korea, Seoul, Republic of Korea; 4 Department of Biological Sciences, Ajou University, Suwon, Republic of Korea; 5 The Anti-Aging Lab, Co. Ltd, Suwon, Republic of Korea

**Keywords:** Organelles, Molecular biology

## Abstract

Zinc (Zn²⁺) is an essential trace element that supports a vast array of cellular processes, including enzymatic catalysis, gene expression, immune regulation and signaling. Its unique redox-inert properties and ability to bind diverse proteins make it indispensable for cellular homeostasis. Zinc is dynamically distributed within cells, where its compartmentalization across organelles, such as the nucleus, endoplasmic reticulum, Golgi apparatus, mitochondria, lysosomes, endosomes and peroxisomes, enables specialized functions crucial for organelle integrity and interorganelle communication. The present Review provides a comprehensive account of organelle-specific zinc homeostasis, highlighting the intricate roles of zinc transporters, metallothioneins and metallochaperones in regulating zinc flux and buffering. Here we discuss how zinc modulates structural and enzymatic processes, stress responses, redox balance and signaling pathways within each organelle. We then provide an integrated overview of how its dysregulation contributes to diverse molecular dysfunctions and pathologies including neurodegeneration, cancer, metabolic disorders and aging. We further examine emerging therapeutic strategies aimed at restoring zinc homeostasis, including supplementation and bioengineered, organelle-targeted delivery systems, as well as advanced tools for visualizing zinc dynamics at subcellular resolution. Together, these insights demonstrate the crucial role of zinc as a compartmentalized regulator of cellular health and a promising target for therapeutic intervention.

## Zinc’s role in cellular health and disease mechanisms

Zinc is a vital trace element essential for numerous biological processes, including enzymatic catalysis, protein stabilization and cellular signaling. This Review explores zinc’s critical roles within cellular organelles and its impact on human health. The authors present an integrated framework detailing how disruptions in organelle-specific zinc regulation can lead to diseases such as neurodegeneration, cancer and metabolic disorders. They use a comprehensive approach, examining zinc’s distribution, transport mechanisms and its role in maintaining cellular homeostasis. Key findings highlight zinc’s involvement in protein folding, redox balance and signaling pathways. The study underscores the importance of precise zinc regulation and suggests potential therapeutic interventions targeting zinc homeostasis. Future research directions include developing organelle-targeted zinc therapies and advanced tools for visualizing zinc dynamics, offering promising avenues for disease treatment and prevention.

This summary was initially drafted using artificial intelligence, then revised and fact-checked by the author.

## Introduction

Zinc (Zn²⁺) is a trace metal that plays a crucial role in cellular function. Its importance to human health was first recognized in the early 1960s, when Iranian patients presenting severe anemia, growth retardation, hypogonadism, skin lesions and lethargy were found to be zinc-deficient^1,2^. Since then, the biological functions of zinc have been increasingly elucidated, particularly at the cellular level, where it is critical for maintaining homeostasis and proper function^3,4^. As the second most abundant transition element in the human body after iron, zinc is essential for a vast range of biological processes, including enzymatic catalysis, structural stabilization of proteins, gene transcription, immune regulation and cellular signaling^5–10^. Its redox-inert nature makes it uniquely suited for roles in oxidative environments, setting it apart from other metal ions such as iron and copper. It is estimated that approximately 10% of the human proteome binds zinc, with zinc finger transcription factors alone accounting for hundreds of proteins that regulate DNA expression and repair^11,12^.

**Fig. 1:**
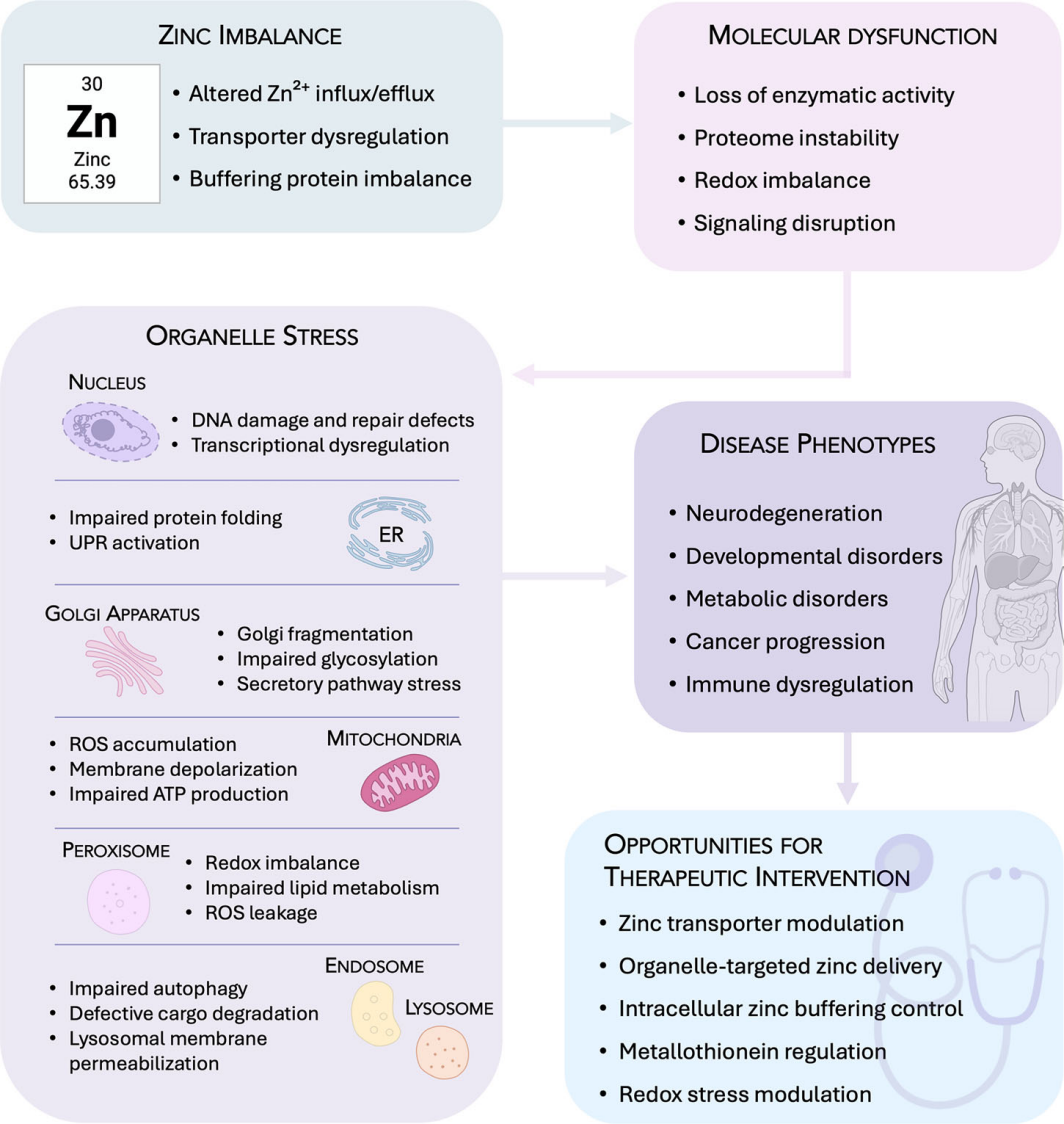
Conceptual framework linking organelle-specific zinc dysregulation to disease. Disturbances in intracellular zinc handling lead to zinc imbalance, triggering core molecular dysfunctions that converge on compartment-specific organelle stress responses. Persistent organelle stress contributes to diverse disease phenotypes. By organizing these events into a causal cascade, the framework highlights mechanistic nodes with potential relevance for future organelle-targeted therapeutic modulation.

Given the essential roles of zinc in the intracellular environment, its precise regulation is of fundamental importance^13,14^. Dynamic compartmentalization across organelles such as the nucleus, endoplasmic reticulum (ER), mitochondria, Golgi apparatus, lysosomes, endosomes and peroxisomes is a requirement for the effective regulation of zinc. In each of these compartments, zinc modulates highly specialized functions. This compartmentalization is maintained by an intricate system of zinc transporters and buffering proteins, which respond to physiological cues and stressors to ensure local zinc availability^15–17^. To contextualize the complexity of these interactions, we present an integrated mechanistic framework (Fig. 1) that summarizes how disturbances in organelle-specific zinc handling propagate across multiple biological layers. Altered Zn²⁺ influx, efflux or buffering leads to intracellular zinc imbalance, which disrupts core molecular processes, including enzymatic activity, protein folding, signaling pathways and redox homeostasis. These molecular defects converge on distinct stress programs in individual organelles, impairing proteostasis, vesicular trafficking, metabolic function or degradative capacity. The accumulation of such organelle stress ultimately contributes to diverse disease phenotypes, ranging from neurodegeneration and metabolic disorders to cancer progression, immune dysregulation and tissue aging. By mapping the cascade from zinc imbalance to molecular dysfunction, organelle stress and disease expression, the framework provides an early overview that anchors the detailed sections that follow and highlights mechanistic points of intervention with potential relevance for future therapeutic modulation^18–21^.

This Review provides a comprehensive and organelle-centered overview of zinc homeostasis and its intracellular dynamics. We begin by summarizing systemic and cellular zinc distribution, emphasizing how zinc is absorbed, buffered, transported and sequestered within specific compartments. We then examine how zinc contributes to specialized organelle functions and, in parallel, how disruptions in these compartmentalized pools initiate cellular stress and dysfunction. In addition, we synthesize current findings on organelle-specific zinc dysregulation and its associated pathological consequences. We further discuss emerging strategies for restoring or manipulating zinc homeostasis, as well as advanced tools for visualizing organelle-level zinc dynamics. Together, these perspectives position zinc as a compartment-integrated regulator of cellular health and identify key opportunities for therapeutic intervention.

## Zinc homeostasis and intracellular dynamics

### Zinc distribution in the body and cell

**Fig. 2:**
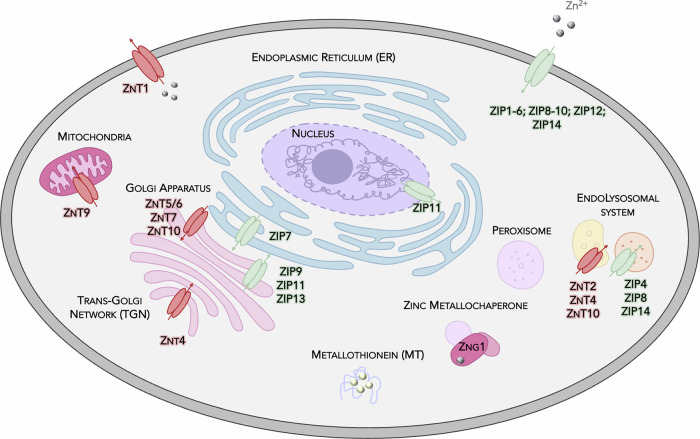
Intracellular distribution of zinc transporters in eukaryotic cells. A schematic illustration of the subcellular localization of zinc transporters from the ZnT (SLC30) and ZIP (SLC39) families across major organelles. The ZnT transporters (red) mediate the zinc efflux from the cytosol into organelles or out of the cell, whereas ZIP transporters (green) promote zinc influx into the cytosol or out of the organelles.

Zinc is a vital trace element distributed widely throughout the human body, with a total content estimated at 1–3 g. The majority is stored in skeletal muscle (~60%) and bone (~30%), with additional pools found in the liver (~5%), skin (~5%), pancreas, kidney and brain^22^. Notably, high concentrations of zinc are also found in the retina and choroid of the eye^23,24^. Unlike elements such as iron or calcium, zinc is not stored in specialized structures and is instead dependent on a dynamic regulation of absorption, transport and excretion. Dietary zinc is absorbed primarily in the small intestine, particularly in the jejunum, where specialized membrane proteins mediate its uptake into enterocytes^25^. Once internalized, zinc can bind to intracellular proteins, be transiently stored or be released into circulation. Zinc transport is mediated by two major protein families, ZIPs (SLC39A) and ZnTs (SLC30A), which are represented in Fig. 2 and will be described in more detail below. In the bloodstream, zinc exists mostly in a protein-bound state, with albumin and α2-macroglobulin serving as its primary carriers^26,27^. Within cells, zinc is distributed among organelles, the cytosol and vesicles in a tightly regulated manner. The total intracellular zinc concentration is estimated to be in the hundreds of micromolar range, yet only a small fraction is exchangeable, with the labile pool maintained at much lower, picomolar levels^28^. Most intracellular zinc is tightly bound to metalloproteins and metalloenzymes, where it serves structural and catalytic roles. By contrast, the labile zinc pool, associated with low-molecular-weight ligands and metallothioneins (MTs), supports signaling and transfer reactions^29^.

### Intracellular zinc trafficking: zinc transporters

Zinc is not uniformly distributed within the cell. Instead, it is compartmentalized across organelles, where it fulfills specialized roles in enzymatic activity, protein folding, oxidative stress regulation and gene expression. This spatial distribution is governed by organelle-localized ZIP (SLC39) and ZnT (SLC30) transporters^17^. The ZIP family facilitates the influx of zinc into the cytosol from either the extracellular space or intracellular organelles, whereas the ZnT family exports zinc from the cytosol to organelles or out of the cell^15,30^. Together, these systems ensure the precise spatiotemporal control of zinc concentrations, preventing both deficiency and cytotoxic excess. Entry into cells occurs predominantly through ZIP family members located on the plasma membrane, which mediate zinc influx by transporting it from extracellular or vesicular compartments into the cytosol. Zinc entry into cells is mediated by several plasma membrane ZIP transporters, which import zinc from the extracellular space into the cytosol. To prevent zinc overload, cytosolic levels are tightly regulated by ZnT1, which serves as the principal efflux pump that exports excess zinc to the extracellular milieu^31^. ZnT10, though classically assigned to Golgi and endosomal compartments, can also traffic to the plasma membrane under specific conditions, such as high extracellular zinc or altered manganese levels, possessing a dynamic role in metal detoxification^32^. In addition, ZnT5 and ZnT6 form heterodimers localized to the Golgi and ER, where they supply zinc to the early secretory pathway for metallation of enzymes such as alkaline phosphatases^33^. ZnT9 is localized to the mitochondria, where it functions as a zinc exporter to prevent mitochondrial zinc overload and maintain metabolic integrity. Finally, a subset of ZIPs localized to intracellular membranes, such as ZIP7 in the ER, and ZIP9, ZIP11 and ZIP13 in the Golgi and ER, support organelle-specific zinc signaling, although their precise subcompartmental localizations remain areas of active investigation. Indeed, although the localization of several zinc transporters is now well established, the localization of others remains uncertain. For instance, the localization of ZIP7 to the Golgi remains debatable. In our previous work, a coexpression analysis of ZIP7 and ZIP13 in fibroblasts showed ZIP7 predominantly in the ER and ZIP13 in the Golgi^34^. Moreover, the depletion of ZIP13 did not induce ER stress, suggesting that its function is distinct from that of ZIP7. These findings indicate that the localization and roles of ZIP7 and ZIP13 require further verification. Moreover, the localization of ZIP9 and ZIP11 to the Golgi/ER remains uncertain, as they lack specific sequence motifs that typically mediate targeting to intracellular compartments^35^. Because the nuclear envelope is structurally distinct from other organelles, evidence for zinc transporter localization there remains limited. The reported presence of ZIP11 in the nucleus is still tentative and requires further confirmation^36,37^. ZnT9 was initially identified as a cytoplasmic and nuclear receptor co‑activator (known as GAC63) on the basis of in vitro assays and bioinformatic predictions^22,38^. However, more recent evolutionary coevolution analyses and microscopy work demonstrate that ZnT9 predominantly localizes to mitochondria and functions as a zinc exporter, with loss leading to mitochondrial zinc overload and dysfunction^39^. Moreover, ZnT10 was initially assigned to the Golgi but later visualized on early endosomes, suggesting a dynamic localization linked to both manganese and zinc export^22^. Together, these coordinated localization patterns ensure that zinc is carefully distributed to support enzymatic functions, signaling pathways and stress responses, while preventing cytotoxic accumulation. Further studies of their intracellular mapping through live-cell imaging and proximity labeling will be essential to clarify remaining uncertainties. In addition to their subcellular localization, ZIP and ZnT transporters also display distinct tissue-specific expression patterns, reflecting the diverse zinc requirements of different organs. These expression patterns are summarized in Table 1.

**Table 1:** Tissue distribution and functional roles of mammalian zinc transporters.

	Transporter	Tissue specificity*	Main role
Zinc importers	ZIP1 (SLC39A1)	Ubiquitous	Basal zinc uptake into cytosol^195^
	ZIP2 (SLC39A2)	Widespread, high in epidermis	Supports epidermal differentiation and turnover^196^
	ZIP3 (SLC39A3)	Widespread, testis, breast	Delivers zinc for mammary epithelial survival and sperm development^197,198^
	ZIP4 (SLC39A4)	Gastrointestinal tract (enterocyte apical), kidney, neurons	Dietary zinc absorption from gut lumen^199^
	ZIP5 (SLC39A5)	Basolateral surface of enterocytes; pancreas, liver, kidney	Zinc retrieval from blood, limits pancreatic toxicity and promotes serosal-to-mucosal recycling^200–202^
	ZIP6 (SLC39A6)	Widespread	Mediates epithelial–mesenchymal transition and cell migration^203^
	ZIP7 (SLC39A7)	Widespread	Releases stored zinc from ER/Golgi to cytosol, activating AKT/ERK, and supports glucose metabolism and insulin signaling^70,204^
	ZIP8 (SLC39A8)	Ubiquitous, especially T cells, lung, testis	Supplies zinc for immune activation and inflammatory signaling^205,206^
	ZIP9 (SLC39A9)	Ubiquitous	Membrane androgen receptor-coupled Zn²⁺ uptake inducing apoptosis^207^
	ZIP10 (SLC39A10)	Brain, liver, kidney, erythroid cells	Zinc uptake in early B-cell development and neuronal cells^208^
	ZIP11 (SLC39A11)	Ubiquitous	Regulation of nuclear zinc homeostasis^37^
	ZIP12 (SLC39A12)	Brain, lung, testis, retina	Supplies zinc for neuronal differentiation, neurite extension and mitochondrial health during development^209,210^
	ZIP13 (SLC39A13)	Ubiquitous	BMP/TGF-β signaling and connective-tissue development^140^
	ZIP14 (SLC39A14)	Liver, heart, pancreas	Regulation of zinc, manganese and non‑transferrin‑bound iron to maintain metal homeostasis^211,212^
Zinc exporters	ZnT1 (SLC30A1)	Ubiquitous	Protects cells by exporting excess zinc to extracellular space^31^
	ZnT2 (SLC30A2)	Mammary gland, pancreas	Loads zinc into secretory vesicles and breast milk^213^
	ZnT3 (SLC30A3)	Neurons (hippocampus, amygdala)	Sequesters zinc into synaptic vesicles for neurotransmission and memory^214^
	ZnT4 (SLC30A4)	Mammary gland, prostate	Delivers zinc to lysosomes and milk^215^
	ZnT5 (SLC30A5)	Ubiquitous	Provides zinc to early secretory enzymes^216^
	ZnT6 (SLC30A6)	Ubiquitous	Forms heterodimers with ZnT5 to metallate ER/Golgi enzymes^217^
	ZnT7 (SLC30A7)	Ubiquitous	Supplies zinc to Golgi-resident glycosyltransferases^78,218^
	ZnT8 (SLC30A8)	Pancreas (pancreatic β-cells)	Loads zinc into insulin granules for hormone crystallization^219^
	ZnT9 (SLC30A9)	Ubiquitous	Exports zinc from mitochondria to maintain respiratory chain activity, prevents mitochondrial dysfunction and acts as nuclear co-activator for nuclear receptors/Wnt genes^157^
	ZnT10 (SLC30A10)	Intestine, liver	High-affinity manganese and zinc efflux to prevent manganese toxicity^220^

*Tissue specificity was assigned on the basis of the highest normalized mRNA expression levels across tissues according to the Human Protein Atlas^221–223^.

### Intracellular zinc storage: MT and zinc metallochaperone

Most intracellular zinc exists in a bound state, with only a small proportion remaining as free, labile zinc. The primary regulators of the labile zinc pool are MTs, low-molecular-weight, cysteine-rich proteins with high zinc-binding capacity^40–43^. MTs bind up to seven zinc ions per molecule via thiolate clusters formed by their 20 cysteine residues, exhibiting high thermodynamic stability and kinetic lability, enabling rapid zinc exchange. These proteins function as intracellular zinc reservoirs, buffering transient fluctuations in zinc availability and releasing zinc in response to oxidative, inflammatory or hormonal stimuli. Furthermore, MTs function as redox sensors; the oxidation of their cysteine residues prompts zinc release, enabling MTs to fine-tune intracellular signaling and defense mechanisms^44,45^. The expression of MT is subject to stringent regulation by zinc status, stress signals and hormones, primarily through metal-response elements in their promoters, with the zinc-sensitive transcription factor MTF-1 serving as an activator. The isoforms MT-1 and MT-2 are expressed in most tissues, whereas MT-3 is primarily expressed in the brain and has recently been associated with bone^46,47^. Moreover, MT-4 has been demonstrated to be associated with hair and skin. In addition to MTs, other cytosolic molecules, such as glutathione and organic acids, contribute to weak zinc binding and help maintain the dynamic equilibrium of the labile zinc pool. Thus, MTs serve not only as buffers and regulators of zinc homeostasis but also as active participants in cellular stress responses. In addition, zinc metallochaperones, such as the recently identified ZNG1^48–50^, facilitate directed zinc delivery to specific enzymes or compartments, though these mechanisms remain under investigation. Together, this tightly regulated buffering system allows cells to maintain zinc availability for signaling and enzymatic functions while avoiding the cytotoxic effects of free zinc. MT and ZNG1 are also included in Fig. 2 for visual reference.

## Organelle-centric zinc regulation in cellular homeostasis and disease

**Fig. 3:**
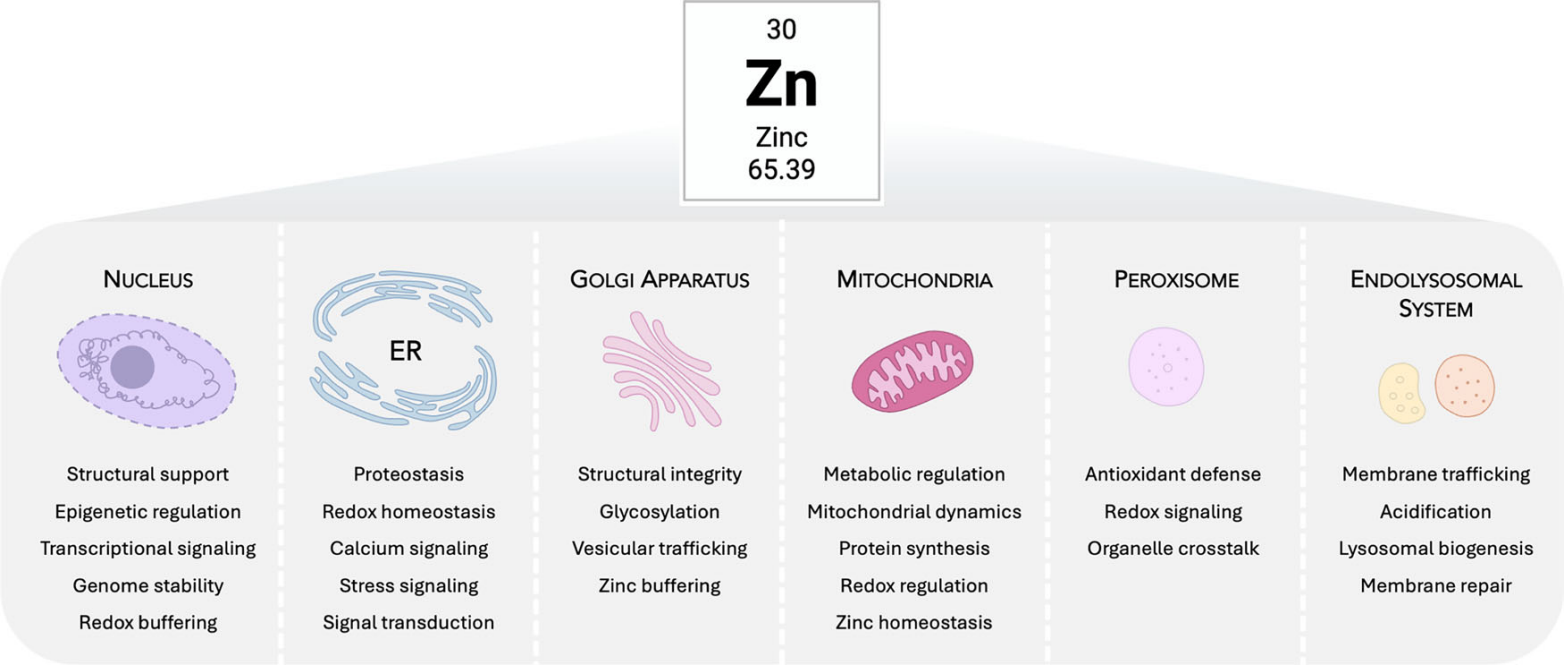
Organelle-centric zinc regulation in cellular homeostasis. Zinc supports essential structural, catalytic and regulatory processes across major intracellular organelles by enabling the function of zinc-dependent proteins, enzymes and signaling pathways. The figure highlights key organelle-specific roles of zinc in maintaining proteostasis, redox balance, metabolic activity, vesicular trafficking and degradative capacity, emphasizing its central contribution to normal cellular function.

Zinc is increasingly recognized not only as a structural cofactor but also as a dynamic signaling ion with organelle-specific roles essential for cellular integrity. Its concentrations are tightly regulated within intracellular compartments to support their diverse functions. The following section explores the compartmentalized roles of zinc across key organelles, illustrating how these distinct intracellular environments depend on zinc for homeostatic control. A schematic overview of these organelle-specific zinc functions is provided in Fig. 3.

### Nucleus

The nucleus is a membrane-bound organelle unique to eukaryotic cells, functioning as the control center of the cell by housing the genetic material and regulating critical processes such as gene expression, protein synthesis, cell growth and division^51^. Zinc constitutes a notable portion of the nuclear metal pool and fulfills several interconnected roles essential for nuclear function. It is estimated that approximately 30–40% of the cell’s zinc is located in the nucleus, whereas the remainder is distributed across the cytosol, organelles, specific vesicles and, to a lesser extent, associated with cell membranes^29^. The nuclear zinc pool comprises both tightly bound structural zinc and a small, dynamic labile fraction that participates in regulatory processes. One of zinc’s primary roles in the nucleus is structural, as it stabilizes zinc-dependent nuclear proteins such as zinc finger motifs and hormone receptors. Zinc finger proteins comprise diverse motifs such as C_2_H_2_, RING, LIM, MYND and PHD domains, which play central roles in transcriptional regulation, chromatin remodeling, DNA repair and RNA metabolism. Similarly, zinc is indispensable for the function of nuclear hormone receptors, which rely on zinc finger motifs for DNA binding and transcriptional regulation^52^.

Zinc also acts as a crucial cofactor for chromatin-modifying enzymes, shaping the epigenetic landscape by modifying histones and methylating DNA. It is essential for the catalytic activity of several classes of histone deacetylases (HDACs), as well as for the structural integrity and function of certain histone acetyltransferases, which together govern chromatin compaction and gene expression, and of DNA methyltransferases (DNMTs), which influence DNA methylation and processes such as lineage commitment and genomic imprinting^11^. In addition to its structural roles, zinc also functions as a regulatory signal within the nucleus. Transient fluctuations in nuclear zinc can modulate chromatin accessibility and redistribute transcription factor binding across the genome. For example, acute zinc shifts reprogram p53 occupancy and alter the expression of its target genes^53^. Similarly, the metal-responsive transcription factor *MTF-1* accumulates in the nucleus upon zinc elevation, binding to metal-response elements to induce *MT* and other protective genes, thus forming a feedback loop that mitigates zinc excess^54,55^.

Beyond these dynamic signaling functions, zinc is indispensable for maintaining genomic stability and regulating cell-cycle progression through its structural integration into nuclear proteins. As a cofactor in the DNA-binding domain of p53, zinc is essential for its tumor suppressor activity^56^. Maintaining nuclear zinc homeostasis requires dedicated mechanisms, including import and buffering systems. So far, ZIP11 is the only zinc importer demonstrated to localize at the nuclear membrane in mammalian cells. It plays a crucial role in shuttling zinc into the nucleus and maintaining nuclear zinc homeostasis^37^. In addition to enzymatic roles, zinc also contributes to nuclear redox homeostasis. MTs, which are highly expressed in the nucleus during oxidative stress, act as both zinc reservoirs and redox buffers^42^. They scavenge free radicals and release zinc in response to ROS, linking antioxidant defense with transcriptional reprogramming.

Zinc shows a multilayered function in the nucleus, ranging from structural support, enzymatic catalysis, epigenetic and transcriptional regulation, signaling, genome maintenance and redox balance. Therefore, maintaining proper nuclear zinc homeostasis is critical for cell survival and homeostasis.

### ER

The ER is a continuous membrane-bound organelle located near the nuclear envelope in the cytoplasm of eukaryotic cells^57,58^. It functions as a central hub for protein synthesis, folding, quality control, lipid metabolism and calcium storage. The ER can be categorized into two distinct functional forms: the rough ER, which is enriched with ribosomes and specializes in protein synthesis, and the smooth ER, which lacks ribosomes and contributes to lipid metabolism and detoxification. The ER lumen provides an oxidizing environment conducive to disulfide bond formation, an essential process in protein maturation.

Within this compartment, zinc plays a crucial role as a structural, catalytic and signaling cofactor. It is required for the function of chaperones such as protein disulfide isomerases and calreticulin, which facilitate disulfide bond formation and ensure proper protein conformation^59^. Zinc also supports ER redox homeostasis by bolstering antioxidant defenses, including MTs and reactive oxygen species (ROS)-detoxifying enzymes such as glutathione peroxidase and peroxiredoxins^60^. As the ER is a major source of ROS during oxidative protein folding, zinc’s antioxidant function is critical to protect protein integrity and maintain proteostasis. Moreover, a key aspect of ER function is calcium storage and regulation^61^. Zinc modulates calcium signaling by influencing the activity of ER calcium-release channels, including the ryanodine receptor (RyR) and the inositol 1,4,5-trisphosphate receptor (IP₃R)^62–66^. Morever, PERK activity depends on zinc-binding domains, and zinc influences eIF2α phosphorylation, a key translational checkpoint. ERp44 also binds zinc through a histidine cluster, which regulates its retrieval of Ero1α and ERAP1 from the *cis*-Golgi back to the ER^67^. Moreover, zinc regulates the expression of BiP, CHOP and other chaperones that buffer misfolded proteins^68^. Through these interactions, zinc fine-tunes calcium-dependent processes such as apoptosis, metabolism and stress responses.

ER zinc homeostasis is maintained by ER/Golgi transporters, including ZIP7, a specific transporter localized to the ER membrane^69^. ZIP7 resides on the ER membrane and releases zinc into the cytosol (following phosphorylation by CK2), thereby acting as a second messenger to activate signaling cascades such as AKT and ERK. ZIP7 also plays a protective role in ER homeostasis because its inhibition triggers ER stress, making it functionally linked to ER-associated degradation (ERAD) and stress mitigation^70^. Therefore, zinc serves as a multifunctional regulator of ER physiology through the maintenance of proteostasis, redox and calcium balance, quality control and signaling.

### Golgi apparatus

The Golgi apparatus is a stacked membrane-bound organelle in the perinuclear region that acts as a center for protein and lipid trafficking, as well as a key player in cargo posttranslational modifications. In addition to these roles, the Golgi regulates several cellular processes, including mitosis, DNA damage responses, stress responses, autophagy, apoptosis and inflammation^71,72^. The Golgi also serves as a transient zinc reservoir, regulating dynamic flux between the cytosol and secretory pathway. Zinc is critical for maintaining the architecture of the Golgi. For instance, zinc acts as a ‘molecular glue’ for Golgi cisternae by binding to stacking proteins such as GRASP55 and Golgin-45^73–75^. Moreover, zinc plays multiple roles in ion buffering, structural maintenance and modulation of stress responses^76^. For instance, Golgi α-mannosidase II (GMII; MAN2A1/MAN2A2) is a Zn²⁺-dependent N-glycan processing enzyme in the Golgi that is required for conversion of hybrid-type to complex N-glycans during N-glycan maturation^77,78^. Zinc can also influence vesicle formation and SNARE-dependent trafficking by contributing to the ionic environment that supports coat assembly and membrane fusion reactions^79^. This is particularly crucial in highly secretory cells such as pancreatic β-cells, where efficient insulin production depends on intact Golgi dynamics. The Golgi apparatus is sensitive to oxidative stress owing to the vulnerability of its lipid-rich membranes and its reliance on tightly regulated redox conditions for glycosylation and trafficking processes^80–82^. Glutathione-dependent redox buffering and Golgi-associated redox enzymes help limit ROS, thereby preserving enzymatic activity and membrane integrity.

As a dynamic and zinc-sensitive organelle, the Golgi apparatus integrates structural, enzymatic and signaling functions that are finely tuned by zinc homeostasis. The abundance of zinc transporters at the Golgi reflects the fine regulation required to maintain its roles in secretion, glycosylation and stress responses.

### Mitochondria

Mitochondria are double-membrane organelles responsible for generating most cellular ATP through oxidative phosphorylation^83^. All known mitochondrial zinc-dependent metalloproteins are synthesized in the cytoplasm and imported into the organelle as unfolded polypeptides, requiring matrix-localized folding and metallation machinery for activation.

Several mitochondrial enzymes depend directly on zinc. The inner-membrane metalloprotease OMA1, which contains a conserved HEXXH zinc-binding motif, cleaves OPA1 to regulate mitochondrial fusion, cristae organization and stress-responsive remodeling of the organelle^84^. In the matrix, the metallo-β-lactamase-family RNase ELAC2 requires zinc for tRNA 3′-end processing, a key step for mitochondrial translation and the assembly of respiratory complexes^85^. The mitochondrial matrix protein Mzm1 also contributes to zinc homeostasis by maintaining labile mitochondrial zinc pools and stabilizing the bc₁ complex (complex III); the loss of Mzm1 leads to reduced mitochondrial zinc and impaired respiratory growth under zinc-limiting conditions^86^.

Zinc further modulates mitochondrial metabolism through its actions on zinc-sensitive dehydrogenases. Both aconitase (ACO2) and the α-ketoglutarate dehydrogenase complex (KGDHC) respond to zinc fluctuations, influencing NADH generation and the efficiency of oxidative phosphorylation^87,88^. In isolated liver mitochondria, zinc reversibly inhibits KGDHC-dependent respiration, demonstrating a mechanistic link between matrix zinc levels and metabolic flux^86^.

Mitochondrial zinc also intersects with redox regulation. MT can localize to the intermembrane space, where its N-terminal β-domain delivers zinc to components of the electron transport chain, modulating respiration in a zinc-dependent and tissue-specific manner^89^.

Zinc trafficking across mitochondrial membranes is mediated by dedicated transporters and carriers. The inner-membrane antiporter ZnT9 exports zinc from the matrix, maintaining intramitochondrial zinc balance^90^. Zinc import is thought to involve the Ca²⁺-activated Mg-ATP carrier SLC25A25 (SCaMC-2), which can transport zinc into the matrix in addition to its canonical substrates^91^.

Together, these mechanisms show that mitochondrial zinc homeostasis is a dynamic, tightly regulated process integrating zinc transport, enzymatic metallation, redox control and metabolic sensing, forming the molecular foundation through which zinc supports mitochondrial structure and function.

### Peroxisome

Peroxisomes are single-membrane organelles central to lipid metabolism and redox homeostasis, responsible for both generating ROS during β-oxidation and detoxifying hydrogen peroxide through catalase and other antioxidant enzymes^92^. Zinc contributes critically to this antioxidant defense as a structural and catalytic cofactor for Cu/Zn-superoxide dismutases (SODs), which convert superoxide radicals into hydrogen peroxide^93^. Both cytosolic and peroxisomal isoforms of Cu/Zn-SOD rely on zinc for stability and activity, and MTs serve as zinc buffers, releasing zinc under oxidative stress to fine-tune redox signaling and enzyme expression^94^. This dynamic zinc flux enables cells to adjust antioxidant capacity, peroxisome proliferation and interorganelle communication.

Peroxisomes and mitochondria also engage in intimate crosstalk to coordinate lipid oxidation and ROS detoxification. Physical tethering between these organelles facilitates metabolite exchange, whereas shared redox signals synchronize adaptive responses^95,96^. When peroxisomal catalase activity is compromised, excess H₂O₂ diffuses into mitochondria, disrupting their dynamics and exacerbating oxidative stress. Zinc supports this interplay by maintaining antioxidant enzymes, including peroxisomal Cu/Zn-SOD and mitochondrial SOD1, and by regulating redox signaling pathways^97^. Collectively, these observations highlight the importance of zinc in maintaining peroxisomal redox balance and functional integrity.

### Endolysosomal system

The endolysosomal system, which includes early and late endosomes and lysosomes, orchestrates receptor recycling, cargo degradation, nutrient sensing and membrane turnover to maintain cellular homeostasis^98^. Within endosomes, zinc contributes to tethering and fusion by stabilizing zinc-dependent tethering factors, such as the zinc finger protein EEA1, which facilitates SNARE complex assembly and ensures efficient cargo sorting and receptor recycling^99,100^.

Zinc also promotes assembly of the vacuolar H⁺-ATPase on endolysosomal membranes, lowering luminal pH to enable ligand–receptor dissociation, enzyme activation and endosome maturation; conversely, zinc deficiency impairs acidification and stalls endosome-to-lysosome trafficking. Beyond regulating V-ATPase activity through assembly, zinc enhances expression of V-ATPase subunits, at least in part via activation of the transcription factor TFEB. The vesicular zinc transporter ZnT2 supports this process by facilitating V-ATPase assembly on lysosomal membranes, thereby promoting lysosomal biogenesis and acidification^101^. Studies in *Caenorhabditis elegans* have shown that the reciprocal regulation of CDF-2 (a ZnT-like importer) and ZIPT-2.3 (a ZIP-like exporter) on lysosome-related organelles mediates zinc storage during excess and release during deficiency, demonstrating a conserved directional flow of zinc^102^. Consistent with this TFEB-dependent remodeling of the endolysosomal system, zinc also induces the expression of lysosomal proteases, including cathepsins B and D, thereby enhancing lysosomal proteolytic capacity^103^. Zinc also regulates the endolysosomal tethering machinery through the HOPS complex, whose VPS18 and VPS41 subunits contain zinc-binding RING domains essential for heterodimer formation and complex stability, which is critical for autophagosome–lysosome and endosome–lysosome fusion^104^. The transient release of zinc via the TRPML1 channel contributes to membrane repair and recovery of lysosomal function after damage, highlighting its role in maintaining organelle integrity^105^.

## Organelle-specific zinc dysregulation and associated pathologies

On the basis of the functional roles outlined in the preceding section, disturbances in organelle-specific zinc homeostasis have been directly linked to molecular dysfunction and a broad spectrum of human diseases. This section consolidates the pathological consequences of zinc dysregulation by systematically linking compartment-specific molecular mechanisms to their associated clinical manifestations, as summarized in Table 2.

**Table 2:** Summary of organelle-specific zinc imbalance mechanisms and pathological consequences.

Organelle	Mechanistic effects of zinc imbalance	Pathological consequences
Nucleus	Dysregulation of zinc-dependent zinc finger proteins and nuclear receptors	Neurodevelopmental disorders^111,112^
	Zinc deficiency disrupts epigenetic regulators including HDACs and DNMTs	Epigenetic instability, aberrant gene expression^113–115^
	Zinc loss destabilizes p53 and DNA repair machinery, promoting genome instability	Impaired DNA repair, genome instability^118^
	Dysregulation of the nuclear zinc transporter ZIP11 causes nuclear zinc accumulation, impairing cell-cycle progression and promoting senescence	Cell-cycle delay, cellular senescence^37^
ER	Zinc deficiency amplifies PERK–eIF2α–ATF4–CHOP-mediated apoptosis during ER stress	Liver injury^123^
	Zinc deficiency induces SOD1 misfolding and impairs ERAD	ALS^124,125^
	Defective UPR and ERAD signaling contribute to ER proteostasis failure	ALS^126–128^
	ZIP7 dysregulation promotes aberrant proliferative signaling	Breast cancer, colorectal cancer^129,130^
	ZIP7-mediated ER zinc release regulates ferroptotic cell death pathways	Breast cancer, renal cancer, kidney ciliopathies^131–133^
	ZIP7 deficiency impairs ER protein folding and connective-tissue development	Connective-tissue defects^69^
Golgi apparatus	Golgi fragmentation and trafficking defects from zinc depletion or transporter loss	ALS, Parkinson’s disease^136^
	Hypoglycosylation from zinc-dependent glycosyltransferase impairment	Congenital disorders of glycosylation^137^
	ZIP13 loss impairs Golgi zinc efflux and collagen biosynthesis	Spondylocheirodysplastic Ehlers–Danlos syndrome^139^
	Overexpression of Golgi zinc transporters supports tumor progression	Cancer (for example, breast, pancreatic, bladder)^146–148^
	ZnT5/ZnT6 deficiency disrupts Golgi glycosylation	Congenital disorders of glycosylation type II^78^
	ZnT7 deficiency limits zinc supply to Golgi metabolic enzymes	Type 2 diabetes, metabolic syndrome^150^
	ZIP13-mediated zinc dysregulation exacerbates tissue injury responses	Myocardial ischemia–reperfusion injury^63,143^
Mitochondria	Zinc-induced mitochondrial ROS generation and membrane depolarization	Cardiomyopathy^151^
	Zinc-dependent STAT3 mitochondrial signaling reduces oxidative stress	Ischemia–reperfusion injury^152^
	Excess zinc impairs neuronal mitochondrial function	Neurodegenerative disorders^153^
	Zinc-regulated mitochondrial transfer supports neuronal recovery	Spinal cord injury^155^
	Mitochondrial zinc accumulation triggers fission and permeability transition	Glaucoma^156^
	Defective mitochondrial zinc efflux via ZnT9	Cerebrorenal syndrome^158^
Peroxisomes	Loss of zinc-binding peroxins impairs peroxisome assembly and protein import	ZSD^159,160^
	Zinc deficiency reduces peroxisomal β-oxidation and lipid catabolism	Metabolic disorders^165^
Endolysosomal system	Impaired lysosomal acidification and reduced autophagic flux	Alzheimer’s, Parkinson’s, ALS^166^
	Zinc activates TFEB and enhances lysosomal biogenesis and proteolysis	Tauopathies/neurodegenerative disorders^103^
	Excess zinc induces lysosomal destabilization and cathepsin leakage	Apoptosis-associated neurodegeneration^168^
	Zinc dysregulation within endolysosomes contributes to cytotoxicity	Breast cancer^169^
	ZnT4 deficiency impairs lysosomal zinc trafficking and secretion	Lethal milk syndrome^170^
	ZIP14 dysfunction disrupts endosomal zinc supply and metabolic regulation	Growth and metabolic disorders^173,174^
	ZIP8 loss impairs endolysosomal metal recycling during inflammation	Iron recycling defects^175^

### Nuclear zinc dysregulation

Given its structural and regulatory roles in the nucleus, the dysregulation of zinc can lead to profound pathological consequences. The dysregulation of zinc-dependent zinc finger proteins and hormone receptors has been associated with various human diseases, including neurodevelopmental disorders and cancer progression^106–112^. Disruption to zinc availability can interfere with the enzymatic functions of HDACs and DNMTs, whose aberrant activity is a feature of epigenetic instability observed in developmental disorders and cancer^11,113–115^. Interestingly, zinc imbalance has been linked to the hypermethylation and silencing of zinc transporters such as ZIP8 and ZIP13, suggesting a feedback loop between zinc status, transporter expression and nuclear epigenetic regulation^116,117^. Furthermore, zinc loss can destabilize the conformation of p53, impairing DNA repair and promoting genome instability^118,119^. Mild zinc deficiency can induce quiescence or stall cells in the S phase of the cell cycle by disrupting DNA synthesis and increasing DNA damage^120^. The disruption of the nuclear transporter ZIP11 causes nuclear zinc to accumulate, impairing cell proliferation, delaying cell-cycle progression and triggering senescence pathways^37^. Together, these findings highlight the pivotal role of nuclear zinc regulation in maintaining genomic integrity, transcriptional fidelity and healthy cell-cycle progression. Together, these findings demonstrate the central role of nuclear zinc homeostasis in preserving genomic integrity, epigenetic stability and proper cell-cycle control.

### ER zinc dysregulation

The ER is highly sensitive to perturbations in zinc homeostasis, and disturbances in luminal or cytosolic zinc can initiate maladaptive stress responses that contribute directly to human disease. When ER zinc balance is disrupted, ER stress pathways become activated, triggering the unfolded protein response (UPR)^121,122^. Although transient UPR activation is protective, chronic zinc deficiency amplifies ER stress signaling, promoting apoptosis and inflammation. Zinc also modulates key nodes within the UPR: PERK activity depends on zinc-binding domains, zinc influences eIF2α phosphorylation and ER chaperones such as BiP and CHOP are regulated by zinc availability^123^. In vivo, zinc deficiency does not independently induce ER stress, yet under pharmacologically induced ER stress conditions, zinc-deficient mice exhibit heightened activation of the pro-apoptotic p-eIF2α–ATF4–CHOP axis, accompanied by apoptosis, steatosis and liver injury. Adequate zinc intake mitigates these effects, partly through the inhibition of PTP1B, showing zinc’s protective role during ER stress.

Beyond canonical UPR activation, zinc deficiency triggers additional maladaptive ER stress mechanisms relevant to neurodegenerative pathology. Under zinc-deficient conditions, wild-type SOD1 undergoes conformational changes resembling mutant SOD1, exposing a Derlin-1-binding region that disrupts ERAD^124,125^. This SOD1-mediated ERAD impairment induces further ER stress, upregulates zinc transporters and suppresses protein synthesis, positioning SOD1 as a molecular sensor that links zinc status to ER proteostasis failure. Defects in both UPR signaling and ERAD have been strongly implicated in neurodegenerative diseases, including Alzheimer’s disease and amyotrophic lateral sclerosis (ALS), highlighting the clinical relevance of maintaining proper ER zinc homeostasis^126–128^.

Perturbations in the ER-localized zinc transporter ZIP7 further illustrate the pathological consequences of ER zinc imbalance. ZIP7 dysregulation has been associated with certain cancers, particularly breast and colorectal cancer, where aberrant ZIP7 signaling contributes to proliferative and survival pathways^129,130^. ZIP7 also acts as a determinant of ferroptosis, linking ER zinc homeostasis to nonapoptotic cell death programs implicated in cancer, such as breast and renal cancer and kidney pathology^131–133^. In addition to these roles, ZIP7 promotes ERAD and, when pharmacologically activated, can reduce ER stress and rescue degeneration in models of protein misfolding^134^. In connective tissue, ZIP7 deficiency impairs protein disulfide isomerase function, leading to defective ER-folding capacity and disrupted development of the dermis, bone and teeth, as demonstrated in connective-tissue-specific ZIP7-knockout mice^69^. These diverse phenotypes show the central role of organelle-specific zinc transport in maintaining ER proteostasis, tissue integrity and cellular resilience across multiple disease contexts.

### Golgi zinc dysregulation

Zinc is essential for maintaining the structural integrity and functional organization of the Golgi apparatus. The disruption of Golgi zinc homeostasis, whether through zinc depletion or transporter dysfunction, leads to unstacking of cisternae, vesiculation and fragmentation of the Golgi, impairing vesicle trafficking and protein sorting^73,135^. Such structural defects are increasingly recognized as contributors to disease. Golgi fragmentation, in particular, has been linked to neurodegenerative disorders including ALS and Parkinson’s disease, where Golgi stress, impaired trafficking and defective processing of secretory proteins exacerbate neuronal vulnerability^136^. Moreover, during zinc deficiency, enzymes such as glycosyltransferases and proteases are impaired, resulting in faulty receptor trafficking, extracellular matrix disintegration and hypoglycosylated proteins, defects that underlie congenital disorders of glycosylation^137^.

Mutations in the Golgi zinc transporter ZIP13 cause spondylocheirodysplastic Ehlers–Danlos syndrome (EDS), a connective-tissue disorder characterized by short stature, hyperextensible skin, joint laxity and skeletal abnormalities^34,35,116,138–142^. Loss of ZIP13 function disrupts zinc efflux from the Golgi, leading to cytosolic zinc depletion, impaired Smad translocation and defective collagen biosynthesis. ZIP13 dysregulation has further been implicated in myocardial ischemia–reperfusion injury and broader connective-tissue pathologies^63,143^.

Other Golgi-localized zinc transporters also contribute to disease. ZnT4 is upregulated in the cerebellum of individuals with Alzheimer’s disease, whereas ZnT6 interacts with amyloid precursor protein to promote amyloid-β aggregation^144,145^. Elevated ZnT5/6 expression has been observed in breast cancer, and increased ZIP11 levels are associated with poor prognosis in pancreatic and bladder cancers^146–148^. Biallelic truncating mutations in ZnT5/ZnT6 similarly lead to congenital disorders of glycosylation type II, presenting clinically with hypoglycosylated transferrin, osteopenia, developmental delay and multisystem involvement^78^. ZnT7, which supplies zinc to early secretory pathway enzymes such as glycosyltransferases and α-mannosidase II, plays key roles in protein maturation and metabolic regulation^149^. ZnT7 deficiency impairs insulin secretion and lipid metabolism, contributing to type 2 diabetes and metabolic syndrome; ZnT7-knockout mice exhibit systemic zinc deficiency, reduced growth, lean body mass and insulin resistance^150^. These defects reveal the centrality of zinc-dependent enzymatic maturation in Golgi-mediated protein quality control.

### Mitochondrial zinc dysregulation

A key consequence of mitochondrial dysfunction is the overproduction of ROS, which damage proteins, lipids, DNA and organelles. Zinc-induced ROS generation and associated toxicity have been demonstrated in various cell types. In rat cardiomyocytes, zinc overload elevates ROS and disrupts mitochondrial membrane potential, triggering PINK1/Parkin-mediated mitophagy and impairing mitochondrial dynamics and biogenesis, with mitofusin 2 (Mfn2) playing a protective role in mitigating zinc-induced mitochondrial damage^151^. In the heart, zinc enhances cardiac mitochondrial function during reperfusion by ERK-dependent phosphorylation of STAT3 at Ser727, which translocates to mitochondria to upregulate ND6 and inhibit succinate dehydrogenase, reducing ROS generation^152^. In neurons, excess intracellular zinc impairs mitochondrial function by dissipating the membrane potential, inhibiting ATP production, increasing ROS generation and permeability transition, disrupting calcium homeostasis and altering mitochondrial dynamics, contributing to neurodegenerative processes^153,154^. Following spinal cord injury, zinc promotes the transfer of healthy mitochondria from microglia to injured neurons by regulating SIRT3-mediated Mfn2, thereby rescuing neuronal mitochondria, reducing oxidative stress, restoring ATP production, enhancing neuronal survival and improving motor recovery^155^. In glaucoma, mitochondrial Zn²⁺ accumulation induces depolarization, increased permeability and fission, including mPTP opening and mitochondrial fragmentation occurring before retinal ganglion cell apoptosis^156^.

Beyond primary mitochondrial disorders, in liver mitochondria, MT localizes to the intermembrane space and can be imported into the organelle, where its N-terminal β-domain delivers zinc to the electron transport chain, inhibiting respiration in a tissue-specific and zinc-dependent manner. Zinc efflux from mitochondria is mediated by ZnT9, a proton-coupled antiporter embedded in the inner membrane. Mutations in ZnT9 have been reported in human patients with developmental defects such as cerebrorenal syndrome^157,158^. Import is thought to be regulated by SLC25A25 (SCaMC-2), a Ca²⁺-activated Mg-ATP carrier that also transports zinc. Precise mitochondrial zinc homeostasis is vital for cellular health owing to its dual roles as a cofactor and a potential toxin, with its dysregulation contributing to various pathologies. Targeting these buffering and transport mechanisms offers promising therapeutic avenues for metal-associated mitochondrial diseases.

### Peroxisomal zinc dysregulation

The disruption of zinc-dependent redox balance in peroxisomes can lead to bioenergetic failure, oxidative damage and disease. Consistent with this, peroxisomal dysfunction is implicated in a wide spectrum of developmental, metabolic and age-related disorders. A prototypical example is the Zellweger spectrum disorders (ZSD), which arise from mutations in PEX genes encoding RING-type zinc finger peroxins such as PEX12 and PEX10, whose zinc-coordinating motifs are essential for peroxisome assembly and protein import^159,160^.

Zinc deficiency further compromises peroxisomal function by impairing the β-oxidation of very-long-chain fatty acids. Reduced zinc availability diminishes the activity of peroxisomal β-oxidation enzymes and downregulates PPARα/γ-mediated transcriptional programs, leading to very-long-chain fatty acid accumulation, oxidative stress and metabolic imbalance^161–165^. These molecular defects may help explain the neurodevelopmental delays characteristic of ZSD and highlight how zinc status modulates lipid catabolism and redox homeostasis. Overall, these defects emphasize the importance of maintaining peroxisomal zinc homeostasis, both through zinc-dependent peroxins and transporter-regulated zinc flux, to sustain lipid metabolism and support proper development.

### Endolysosomal zinc dysregulation

Defects in the endosome–autophagosome–lysosome pathway, the major machinery for degrading protein aggregates and damaged organelles, are central drivers of neurodegenerative diseases such as Alzheimer’s disease, Parkinson’s disease and ALS^166^. Impaired acidification, fusion defects and reduced endosome–autophagosome–lysosome pathway flux compromise the clearance of pathogenic aggregates, and raising cellular cAMP or free zinc has been proposed as a strategy to restore lysosomal pH and proteolytic capacity in affected neurons^166^.

Zinc supplementation activates TFEB, a master regulator of lysosomal biogenesis, and improves autophagic flux in neuroblastoma cells expressing wild-type or mutant tau^103^. In parallel, zinc rapidly induces the expression and activation of lysosomal proteases cathepsin B and D, through early V-ATPase-dependent acidification and later TFEB-mediated transcription. These coordinated responses promote TFEB nuclear translocation, enhance lysosomal and autophagic gene expression more effectively than rapamycin and attenuate phosphorylated tau, total tau and p62 accumulation, highlighting zinc’s therapeutic potential in restoring lysosomal proteolysis and autophagy^103,167^. However, under zinc excess, lysosomal destabilization and leakage of cathepsin D into the cytosol can trigger mitochondrial damage and apoptosis via the lysosome–mitochondria axis^168^. Zinc dysregulation within the endolysosomal system also intersects with cancer biology: the alcohol-deterrent and emerging anticancer agent disulfiram disrupts endolysosomal structure and raises intraluminal zinc in breast cancer cells, implicating zinc dysregulation in its cytotoxicity and suggesting that extracellular zinc availability modulates its efficacy^169^.

Transporter dysfunction within the endolysosomal system leads to diverse systemic phenotypes. ZnT4-deficient mice exhibit the classic ‘lethal milk’ phenotype, in which mammary zinc secretion fails and offspring perish without supplementation^170–172^. ZIP14 localizes to both the plasma membrane and endosomes, where it transports zinc and other metal ions at acidic pH. In hepatocytes, ZIP14 translocates from the plasma membrane to endosomes during glucose uptake, supplying zinc required for endosomal protease activity and insulin receptor regulation. ZIP14-knockout mice develop dwarfism, osteopenia, impaired skeletal growth, ER stress, metabolic abnormalities and manganese overload, illustrating the broad physiological impact of endosomal metal transport^173,174^. In addition, ZIP8-knockout mice display impaired iron recycling during inflammation, elevated splenic iron and reduced serum iron, highlighting ZIP8’s role in systemic metal handling^175^. These diverse phenotypes demonstrate the essential role of endolysosomal zinc homeostasis in maintaining neuronal proteostasis, systemic metal balance and tissue integrity across multiple disease contexts.

## Organelle-associated therapeutic targeting of zinc dysregulation

Zinc’s compartmentalized functions within cellular organelles highlight its potential relevance to therapeutic strategies. By selectively addressing zinc imbalances in specific subcellular compartments, emerging strategies seek to restore cellular homeostasis across various disease contexts. Although the importance of zinc in health and disease has been recognized since the 1960s, organelle-targeted zinc therapies remain an evolving and largely unexplored field. In the following section, we highlight recent advances in therapeutic approaches that exploit zinc biology, including studies that use zinc as a pharmacological modulator of intracellular organelles and nanotechnology-based zinc materials to mitigate organelle stress. Numerous clinical trials on zinc supplementation have been recently conducted or are ongoing^176,177^, but these are not organelle-specific and thus fall outside the scope of this Review.

### Zinc supplementation and pharmacological modulation

Although zinc supplementation has long been recognized to correct systemic and cellular zinc deficits, recent studies have specifically highlighted its ability to modulate organelle homeostasis. These recent findings merit closer attention, as they reveal mechanistic insights and therapeutic potential beyond the traditional view of zinc as a general micronutrient. Classical zinc supplementation remains a widely explored approach to correct cellular zinc deficits. Beyond systemic effects, recent studies suggest that zinc can directly alleviate subcellular stress by restoring organelle-specific zinc levels, particularly in the ER and mitochondria. For example, zinc treatment has been shown to attenuate ER stress in porcine oocyte maturation by upregulating ZIP14 and ZIP10 and restoring redox homeostasis, ultimately improving developmental outcomes^121^. Similarly, in hepatocytes exposed to lipotoxic stressors, zinc supplementation reduces cytotoxicity by mitigating ER stress and enhancing antioxidant defense, demonstrating its capacity to preserve proteostasis and limit inflammatory responses^178^. Moreover, zinc was shown to promote mitochondrial function through SIRT3–Mfn2-mediated pathways in microglial–neuron systems, indicating the precise modulation of organellar metabolism^155^. Moreover, zinc supplementation has demonstrated the ability to enhance lysosomal function by activating TFEB-mediated lysosomal biogenesis and promoting autophagic flux^103^. These findings indicate that targeted zinc administration can modulate organelle integrity, signaling pathways and metabolic functions in multiple compartments. However, several zinc-related pathways may not properly respond to zinc repletion. For instance, in our recent study, zinc supplementation increased MT-1 expression yet failed to restore Golgi protein expression during Golgi fragmentation^73^. This suggests that zinc deficiency and organelle dysfunction can become uncoupled under certain conditions, demonstrating the complexity of zinc’s role in organelle biology.

### Nanotechnology and bioengineered zinc delivery systems

As our understanding of zinc’s role in organellar biology deepens, so does the demand for targeted delivery systems capable of modulating zinc flux with high spatiotemporal precision. Advancements in nanotechnology and bioengineering have enabled the development of smart delivery platforms capable of targeting zinc to specific cells or organelles. These systems aim to overcome the limitations of conventional supplementation by achieving more precise control over zinc localization and timing, paving the way for highly tailored therapies with reduced systemic side effects. For instance, engineered zinc-based nanoparticles such as zinc ferrite cores conjugated with disease-targeting moieties have been investigated for their ability to selectively deliver zinc into inflamed or dysfunctional cells, triggering organelle-specific responses. Fibroblast activation protein (FAP)-targeted zinc ferrite nanoparticles accumulated in FAP-expressing synovial fibroblasts in a rheumatoid arthritis model by activating ER stress and mitochondrial dysfunction, resulting in controlled cytotoxicity in inflamed joints, sparing healthy tissue^179,180^. Similarly, in oncology, zinc-based nanomaterials have been used to induce ER stress and the UPR, culminating in immunogenic cell death and enhancing the efficacy of immune checkpoint blockade therapies^181,182^. Recent innovations also exploit the intrinsic interactions of zinc-based nanomaterials with intracellular organelles to enhance therapeutic delivery and monitoring. For instance, a zeolitic imidazolate framework (ZIF-8)-derived carbon dot system (ZCD) was engineered to carry doxorubicin and respond to the acidic tumor microenvironment through hierarchical size and charge transformations^183^. Upon accumulation in solid tumors, ZCD disassembled into smaller, neutrally charged particles that were endocytosed into lysosomes and further transformed into positively charged species capable of targeting the Golgi apparatus. This lysosome-to-Golgi trafficking enabled deep penetration into tumors via Golgi-mediated transcytosis, enhancing anticancer efficacy. Notably, the carbonized ZCD also exhibited pH-activated fluorescence, allowing the real-time monitoring of penetration depth. These bioengineered systems represent a convergence of materials science and cell biology, marking a transition from generalized zinc supplementation to designer therapies that exploit zinc-mediated organelle stress for clinical benefit.

## Tools for intracellular zinc detection

The accurate mapping of zinc within intracellular compartments is crucial for understanding its regulatory functions in organelle function, signaling and homeostasis. A range of tools has been developed to detect intracellular zinc. Among these, fluorescent small-molecule probes and genetically encoded sensors have become the most widely used and practically applicable tools for studying labile zinc dynamics within organelles of live cells. They offer the required combination of sensitivity, spatial resolution, organelle targeting and compatibility with live-cell imaging. By contrast, other techniques such as mass spectrometry-based imaging, synchrotron X-ray fluorescence and electron microscopy with elemental detectors are powerful for quantifying total zinc and mapping its distribution in fixed specimens at the nanometer resolution. Although these methods provide valuable structural and compositional insights, they are less suitable for measuring zinc dynamics in organelles (Table 3). Therefore, in this Review, we focus on advances in fluorescent probe technologies, which remain the gold standard for dynamic, organelle-specific imaging of labile zinc in live cells.

**Table 3:** Summary of tools for cellular zinc detection.

Category	Read-out	Sample type	Principal strengths	Key limitations
Fluorescence probes	Labile zinc/real-time	Live	Dynamic/organelle-specific/multiplexing	Poor selectivity versus other metals; photobleaching; only labile zinc; not total zinc^189,224,225^
Mass spectrometry (secondary ion mass spectrometry, laser ablation inductively coupled plasma mass spectrometry)	Total zinc/isotopes	Fixed	High spatial resolution (nanometer for nano-secondary ion mass spectrometry)/quantitative/isotope tracing possible	Destructive; poor zinc ion yield (secondary ion mass spectrometry); no speciation; complex prep; fixed samples^226–228^
X-ray imaging	Total zinc/chemical speciation	Fixed (often cryo)	High sensitivity/chemical state information (speciation)/≤50–100-nm spatial resolution	Requires synchrotron; time-intensive; limited sample throughput^229–232^
Electron microscopy (scanning transmission electron microscopy–energy dispersive X-ray spectroscopy)	Total zinc/ultrastructure	Fixed/ cryo	Nanometer-scale resolution/structural and elemental maps correlated	Low zinc sensitivity; not very quantitative; expensive; technically demanding; destructive^233^

### Fluorescent probes for intracellular zinc detection

Fluorescence-based sensors are the most widely used tools for monitoring intracellular zinc, particularly the labile zinc pool in living cells. These approaches exploit the property of fluorophores to change their emission upon binding zinc, enabling the real-time, subcellular visualization of zinc dynamics^184^. Small-molecule probes, such as TSQ and Zinquin, pioneered live-cell imaging in the 1990s, although early designs were limited by poor selectivity and lack of organelle specificity^185,186^. Over the years, probe design evolved to include more selective and photostable fluorophores, near-infrared and two-photon probes for deep-tissue imaging and organelle-targeted sensors directed to mitochondria, Golgi, nucleus or ER. Genetically encoded sensors emerged in the early 2000s as an alternative platform for ratiometric, reversible and organelle-specific zinc imaging. Pioneering designs such as eCALWY and ZapCY exploit fluorescence resonance energy transfer between two fluorescent proteins bridged by a zinc-binding domain^62,187^.

Recent innovations aim to overcome earlier limitations and improve the spatial and temporal resolution of intracellular zinc detection. For instance, zinc superresolution targeted imaging with minimal overlap has emerged as technology that integrates structured illumination microscopy with specially designed fluorophores that selectively localize to distinct organelles, achieving sub-100-nm resolution while minimizing spectral overlap^188^. To address organelle-specific pH and redox environments, turn-on zinc fluorescent probes (ZnDA-2H and ZnDA-3H) with low pH sensitivity and high affinity were developed and targeted to the cytosol, nucleus, ER and mitochondria using HaloTag technology, enabling the precise quantification of labile zinc distribution^189^. For the ER specifically, a theranostic Ir(III) complex (Ir-ER-Zn) that combines zinc-responsive phosphorescence with ER targeting has been developed to monitor zinc dynamics during immunogenic cell death while inducing ER stress and enhancing antitumor immunity^190^. For the Golgi apparatus, a small-molecule probe using a trityl-protected cysteine motif was developed to selectively image mobile zinc under physiological and oxidative stress conditions^191^. More recently, a ratiometric fluorescence nanosensor (Golgi-Zn) with high sensitivity, selectivity and robust pH stability enabled the quantitative monitoring of zinc in the Golgi, revealing that nanoplastics exposure increases Golgi zinc levels and links zinc homeostasis to nanoplastic-induced stress^192^. In addition, a small-molecule probe, ZnDA-1H, was developed with low pH sensitivity and high zinc selectivity for targeting the Golgi. Using this probe, the zinc concentration in the Golgi of HeLa cells was estimated at 25 ± 1 nM, supporting a role for labile zinc in secretory pathway regulation^193^. As nuclear zinc is predominantly present as tightly bound, structural zinc associated with proteins, it is not detectable by fluorescent indicators, which only report on the chelatable, loosely bound zinc fraction^194^. Together, these advancements have provided powerful tools to visualize zinc dynamics with high spatial and temporal resolution at the organelle level, deepening our understanding of zinc’s compartmentalized roles in cellular physiology and pathology.

## Future perspectives

Collectively, the preceding sections highlight zinc as a tightly regulated, compartmentalized regulator of cellular homeostasis whose disruption contributes directly to human disease. Despite this mechanistic insight, the clinical assessment of zinc status remains largely confined to systemic measurements, which poorly reflect intracellular distributions or organelle-specific dysfunction. Bridging this gap represents a critical translational challenge and an opportunity to refine both diagnostic and therapeutic strategies.

**Fig. 4:**
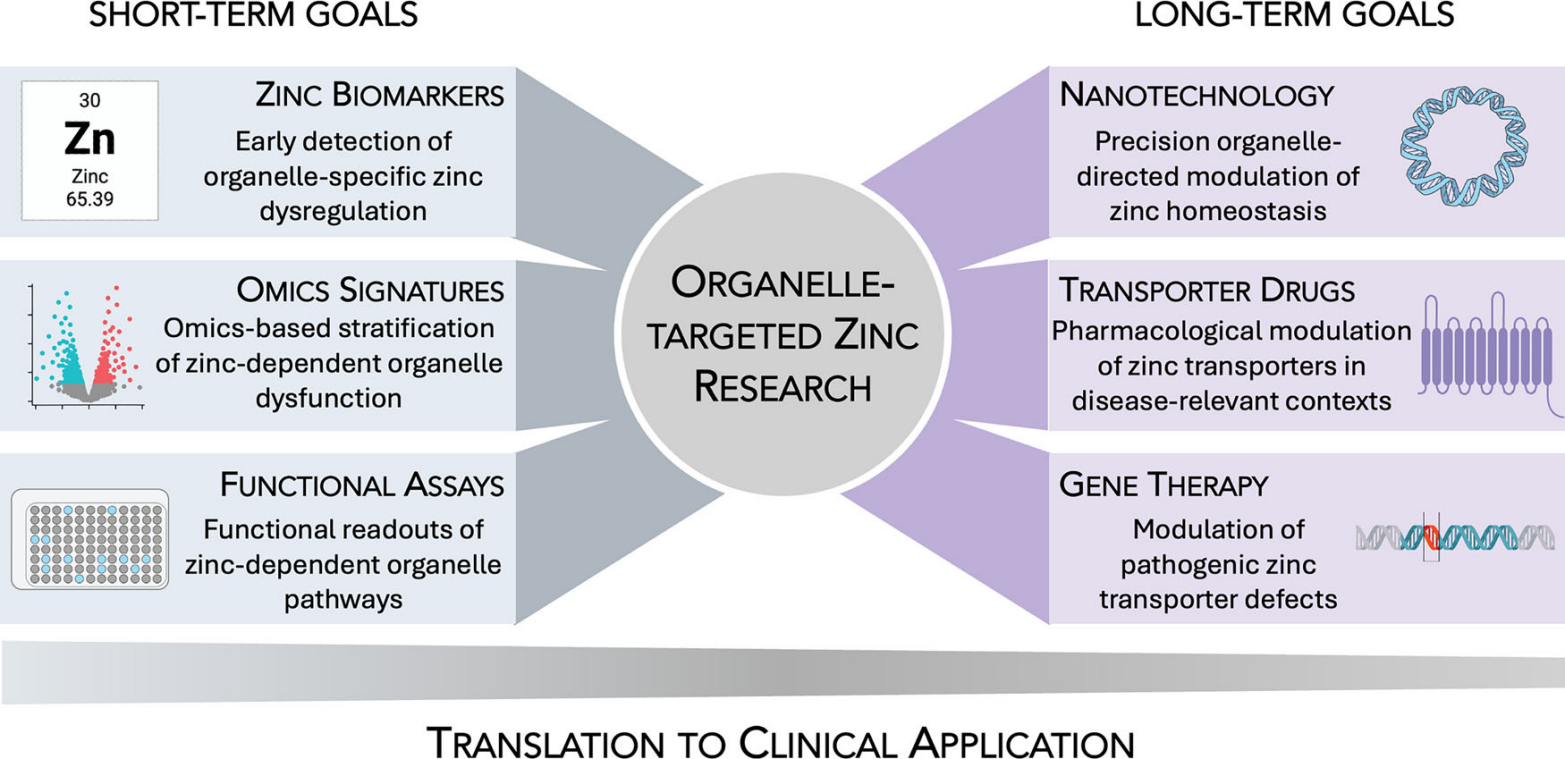
Future translational opportunities in organelle-targeted zinc research. The schematic outlines short- and long-term research directions required to translate mechanistic insights into zinc compartmentalization toward clinical application. Short-term priorities emphasize the development of biomarkers, omics-based signatures and functional assays to detect organelle-specific zinc dysregulation in vivo, whereas long-term goals focus on organelle-directed delivery platforms, transporter-selective modulation and gene-based strategies. Together, these stages define a translational trajectory from fundamental zinc biology to precision intervention.

As summarized in Fig. 4, short-term priorities focus on improving the clinical interpretability of organelle-specific zinc dyshomeostasis in vivo, whereas long-term efforts aim to move beyond generalized supplementation toward interventions capable of modulating zinc handling with subcellular precision.

### Short-term priorities: biomarkers of organelle-level zinc dyshomeostasis

A major limitation in current clinical practice is that circulating zinc concentrations provide little insight into intracellular zinc distribution or the functional state of zinc-dependent organelles. Short-term translational progress will therefore depend on developing diagnostic strategies that more directly capture the downstream consequences of organelle-specific zinc imbalance, even if indirect.

#### Translational imaging and sensing approaches

The continued development of zinc-sensitive probes and imaging modalities that move beyond purely experimental systems toward clinically compatible readouts will be essential. Although the direct quantification of organelle zinc pools in patients remains unrealistic, advances such as organelle-biased tracers, ratiometric reporters and imaging strategies adaptable to tissue or biopsy specimens may enable spatially resolved assessment of zinc dyshomeostasis in vivo.

#### Accessible multiomics signatures of zinc-dependent organelle dysfunction

Organelle-specific zinc imbalance produces characteristic transcriptional, proteomic and metabolomic consequences that may be detectable in accessible biospecimens such as blood, cerebrospinal fluid or urine. Identifying and validating such signatures could enable the scalable, indirect monitoring of zinc-dependent organelle stress, analogous to how mitochondrial or ER stress biomarkers are currently used clinically.

#### Functional pathway readouts as practical proxies

Assays that quantify the activity of zinc-sensitive organelle pathways, such as secretory pathway processing, glycosylation capacity, UPR activation or lysosomal proteolysis, may provide clinically actionable proxies for luminal zinc availability and transporter performance. Although not measuring zinc directly, such functional readouts may offer greater relevance for disease stratification and therapeutic monitoring.

#### Long-term priorities, organelle-targeted zinc modulation

The central therapeutic goal is to progress beyond nonspecific systemic zinc supplementation toward interventions that modulate zinc handling within defined subcellular compartments in a disease-context-dependent manner.

### Organelle-directed delivery platforms

Engineering delivery systems capable of subcellular targeting and controlled zinc release remains a major challenge. Nanotechnology-based platforms and bioengineered carriers offer promising routes to concentrate zinc or zinc-modulating agents within specific organelles while minimizing off-target redistribution and systemic toxicity.

### Transporter-selective pharmacological modulation

Growing structural and functional insight into zinc transporters raises the possibility of developing small molecules that selectively modulate individual transporters or their regulators. Such approaches could enable the fine-tuned correction of organelle-specific zinc flux without broadly altering total cellular zinc levels.

### Gene- and proteostasis-based strategies for monogenic disorders

For diseases driven by single-transporter defects, gene-based therapies and pharmacological chaperones that restore proper trafficking, stability or function of mutant zinc transporters represent plausible longer-term strategies. These approaches align with emerging precision medicine paradigms and may offer the durable correction of organelle-specific zinc dysregulation.

## Conclusion

Zinc functions as a unifying regulator of organelle physiology, supporting genome stability, protein folding, metabolic balance, vesicular trafficking and degradative capacity. Although individual organelles rely on zinc in distinct ways, these roles are coordinated through an interconnected network of transporters, MTs and emerging metallochaperones that dynamically redistribute zinc in response to cellular demand.

The disruption of this network produces organelle-specific imbalances that propagate into cellular dysfunction and contribute to a wide spectrum of human diseases, including neurodegeneration, cancer, metabolic disease and developmental disorders. Importantly, these findings provide a mechanistic explanation for the limited diagnostic value of circulating zinc measurements and the variable clinical efficacy of nonspecific zinc supplementation, which fail to capture or correct compartment-resolved zinc dysregulation.

Viewed through an organelle-centric lens, zinc-related pathologies are better understood as disorders of intracellular zinc handling rather than uniform states of deficiency or excess. This conceptual shift has direct clinical implications, reframing zinc dysregulation as a problem of localization, transport and pathway-specific vulnerability. Recognizing zinc homeostasis as an organelle-resolved process therefore provides a more accurate framework for interpreting disease mechanisms and therapeutic responses and for integrating zinc biology into precision approaches to diagnosis and treatment.
